# Two Cases of Nonsuicidal Self-Injury Comprising Partial Autoamputation of the Apex of the Tongue

**DOI:** 10.1155/2020/8691270

**Published:** 2020-02-21

**Authors:** Takashi Moriya, Hitoshi Sato, Kenichi Takeda, Kaori Ikezaki, Ryogo Katada, Tatsuo Shirota

**Affiliations:** ^1^Department of Oral and Maxillofacial Surgery, Division of Oral Oncology, Showa University School of Dentistry, Tokyo, Japan; ^2^Department of Oral and Maxillofacial Surgery, Showa University, School of Dentistry, Tokyo, Japan

## Abstract

The prevalence of nonsuicidal self-injury (NSSI) in adults is lower than that in adolescents and it is more prevalent in patients with psychiatric disorders. Sleep disturbances such as nightmares are associated with NSSI after accounting for depression; thus, persons with major NSSI sometimes present at medical institutions during the night seeking emergency treatment. Gingival tissues comprise the most frequent target of self-injury of the oral cavity using oral hygiene tools. Most NSSI in the oral cavity is minor because such tools are blunt. Major NSSI such as autoamputation of the tongue is rare. We describe two patients who partially autoamputated the apex of their own tongues using edged tools. Case 1 was a 55-year-old female with depression who had defaulted from psychiatric intervention. She had cut off her tongue using a Japanese kitchen knife and presented with the dry, necrotic amputated portion and blood oozing from the remainder of her tongue. We debrided and sutured the remainder of the tongue without reattaching the amputated portion. Her postoperative course was uneventful, and she was free of adverse events such as functional disability and wound infection. Case 2 was a 69-year-old female with schizophrenia who had defaulted from psychiatric intervention and had cut off her tongue using scissors. The amputated portion of the tongue was lost and the remainder, which was oozing blood, was debrided and sutured. She defaulted on a follow-up appointment. Neither of these patients had suicidal intent. The prevalence of NSSI across all age groups has recently increased, and the risk that self-injury will become normalized has become a concern. Thus, dentists as well as oral and maxillofacial surgeons should be aware of the possibility that patients will present with major NSSI requiring emergency treatment.

## 1. Introduction

Nonsuicidal self-injury (NSSI) is usually described as the deliberate, direct destruction or alteration of body tissue without conscious suicidal intent [1, 2], and it is a unique risk factor for suicide [3]. Nonsuicidal self-injury occurs among 17.2% of adolescents, 13.4% of young adults, and 5.5% of older adults [4]. It often occurs in patients with psychiatric disorders such as schizophrenia [5–7], autism [8], and depression [9], and the target of NSSI is often the skin, eyes, or genitalia [10]. Much of the described NSSI in the oral cavity has been minor, because the gingival tissues are the most frequent site of injuries and the tools have usually been toothbrushes and brush picks [11–15]. On the other hand, major NSSI is a considerable public health problem because it is also associated with high rates of emergency room utilization [16]. We describe two adult patients who presented at our emergency room during the night with partial autoamputation of the apex of the tongue.

## 2. Case Reports

### 2.1. Case 1

**Figure 1:**
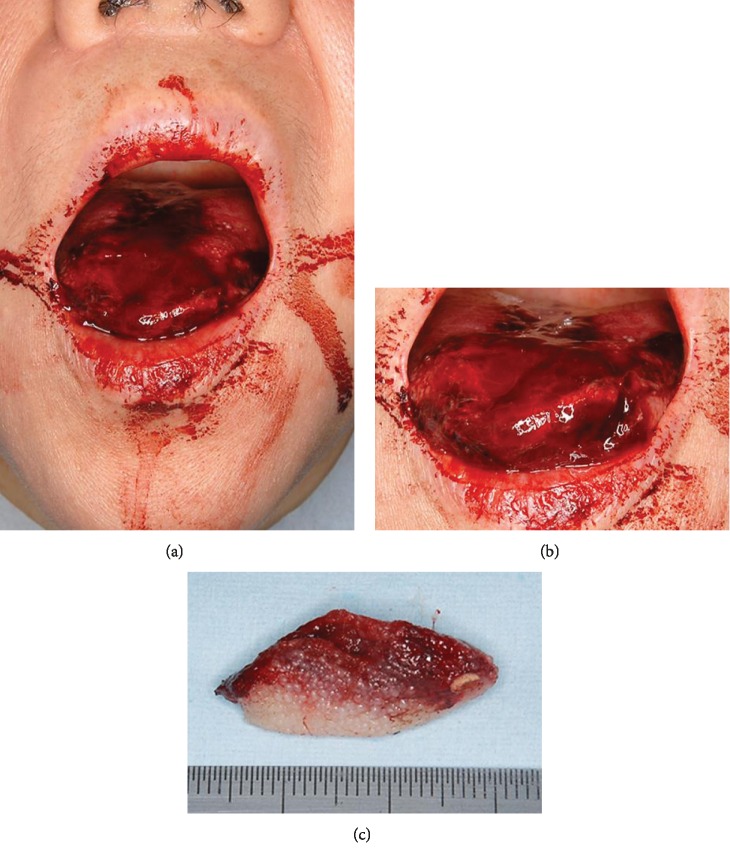
Case 1: preoperative images of the oral cavity. At rest (a), tongue protrusion (b), and amputated portion (c).

**Figure 2:**
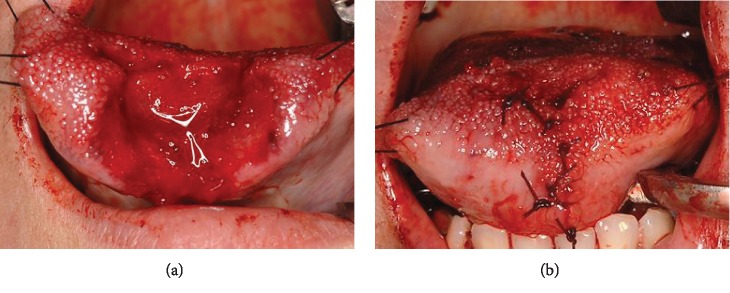
Case 1: operative images of the oral cavity. After debridement (a) and suture (b).

A 55-year-old Japanese female presented in November 2011 having autoamputated the apex of her tongue with a Japanese kitchen knife. She had chronic depression but had defaulted from psychiatric intervention and thus was not under prescribed medication for depression. She had endured parasomnia with nightmares for several months. She had cut her tongue after experiencing a terrible nightmare about her son and presented at the emergency room eight hours later at 05:00 hours. Her vital signs were unremarkable, but blood continuously oozed from the remaining part of the tongue (Figure 1(a)). No other lacerations were evident, and the remainder of the tongue had normal mobility (Figure 1(b)). She had also brought the dry and necrotic amputated portion (2.8 × 1.5 cm) (Figure 1(c)). Active bleeding from the lingual arteries was not evident. A bite block was inserted between the posterior molars, and then we debrided (Figure 2(a)) and sutured the remainder of the tongue using an antimicrobial Vicryl® Plus suture coated with polyglactin 910 and triclosan (Ethicon Inc., Somerville, NJ, USA) under local anesthesia with xylocaine 2% and adrenaline (1 : 80,000 dilution) (Figure 2(b)). The amputated portion of the tongue was not reattached. We prescribed loxoprofen sodium hydrate (Loxonin; Daiichi Sankyo Co. Ltd., Tokyo, Japan) 60 mg for pain control and the antibiotic amoxicillin hydrate (Sawacillin; Astellas Pharma Inc., Tokyo, Japan) 3 × 250 mg daily to prevent infection. We provided the patient with postoperative instructions regarding oral hygiene, a soft diet, and smoking cessation and also explained the importance of resuming psychiatric treatment for chronic depression. Her postoperative course was uneventful, and she has remained free of functional disability and wound infection.

### 2.2. Case 2

**Figure 3:**
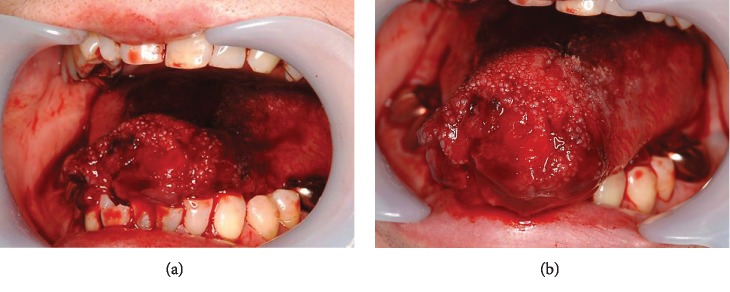
Case 2: preoperative images of the oral cavity. At rest (a) and tongue protrusion (b).

**Figure 4:**
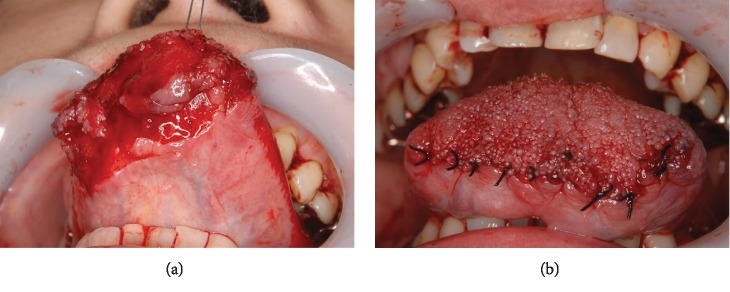
Case 2: operative images of the oral cavity. After debridement (a) and suture (b).

A 69-year-old Japanese female presented in January 2011 having autoamputated the apex of her tongue with scissors. She had a 10-year history of schizophrenia but had defaulted from psychiatric intervention and stopped taking prescribed medication. She also had parasomnia with arousal disorders. She arrived at the emergency room three hours after the autoamputation at 03:00 hours. Her vital signs were stable. Although blood oozed from the major portion of the tongue, the lingual arteries were not damaged (Figure 3(a)). No other lacerations were evident, and the mobility of the remaining portion of the tongue was normal (Figure 3(b)). The amputated portion was missing; thus, the remainder of the tongue was debrided and sutured using Vicryl® Plus antimicrobial coated sutures (Ethicon Inc.) under local anesthesia (xylocaine 2% with adrenaline (1 : 80,000 dilution) (Figures 4(a) and 4(b)). The patient was prescribed with loxoprofen sodium hydrate (Loxonin; Daiichi Sankyo Co., Ltd.) for pain control and amoxicillin hydrate (Sawacillin; Astellas Pharma Inc.) to avoid infection. We provided the patient with postoperative instructions including oral hygiene and a soft diet. We also described the importance of resuming psychiatric treatment for schizophrenia and follow-up with us. However, this patient did not keep her first postoperative appointment and was lost to follow-up.

## 3. Discussion

The prevalence of NSSI has recently increased in both sexes and across age groups in England [17], and the risk that self-mutilation will become normalized has become a concern. Although NSSI is more prevalent among young women and girls in Western countries [17], it is more prevalent among male than female Chinese college students [18]. This prevalence in China is remarkable in rural, but not urban areas [18, 19]. This might be related to coping method with emotional stress that is influenced by culture and gender norms. Since a history of NSSI is a clinical marker for subsequent suicide attempts [3], self-mutilation to cope with emotional stress could have serious long-term public health implications.

Major NSSI are generally associated with severe mental illness [5–9, 20–22] and often result in the permanent loss of organs such as the skin, eyes, genitalia [10], or their functions [21–23]. Davies and Doyle have identified a close association between psychiatric disorders and major NSSI because at least 79.3% of patients with major NSSI also have chronic psychiatric disorders [22]. Some individuals are believed to practice NSSI because of feeling overwhelming guilt, depersonalization, or experiencing hallucinations [1, 24]. Since the degree of NSSI is related to anger, anxiety, or impulsivity [25], nonsuicidal autoamputations are rare [26]. On the other hand, disruptions in sleep are associated with an increased tendency towards emotional dysregulation [27]. Nightmares rather than insomnia symptoms have recently been associated with NSSI after accounting for depression [28]. One of our patients (Case 1) mutilated herself with a knife after a dreadful nightmare about her son, about whom she felt overwhelmed with guilt as he was a juvenile delinquent. However, she had no suicidal intent. The other patient (Case 2) had often experienced hallucinations during arousal from sleep. Thus, sleep arousal disorders and schizophrenic hallucinations might have led her to the autoamputation even though she also had no suicidal intent. Since both these patients had defaulted from psychiatric intervention, relapsing symptoms of their psychiatric disorders might have been involved in their NSSI. Moreover, dentists as well as oral and maxillofacial surgeons should be aware that patients with major NSSI associated with sleep disturbances such as nightmares might present late at night seeking emergency treatment.

Minor NSSI do not lead to major disabilities and are relatively common, whereas major NSSI such as autoamputations can lead to significant disability. Most NSSI of the oral cavity are caused using tools of oral hygiene such as toothbrushes or brush picks, and gingival tissue is a frequent target [11–15]. Thus, most NSSI in the oral cavity such as erosion, ulcers, or periodontal clinical attachment losses are minor. To determine the characteristics of intraoral NSSI using edged tools, we searched the PubMed database in June 2019 using the search terms “self-harm” OR “self-injury” OR “NSSI” OR “self-mutilation” OR “self-inflicted injuries” AND “intra oral” OR “oral cavity” OR “tongue” OR “gum” OR “gingiva” OR “lip” OR “teeth.” Data were screened in two steps. The first excluded articles with incomplete text or with missing data. The second step involved filtering by patient age, sex, medical history, tools used for mutilation, and suicidal intent.  Table 1 describes five patients with NSSI of the oral cavity that they inflicted using edged tools [20, 21, 29]. As far as we are aware, only three patients who have used edged tools to inflict intraoral NSSI have been described. The mean age of these five patients (including the two described herein) was 46.8 ± 21.0 years, and three were female. Two patients used scissors and three used knives. Three of the major NSSI involved tongue amputations and were associated with schizophrenia or depression.

Treating physicians are faced with challenging decisions when NSSI involves amputation, because some evidence has shown that good rehabilitation and motivated patients are needed to obtain favorable functional outcomes after replantation [20, 21, 30]. Hong and Eun have described successful replantation of an amputated tongue after NSSI in a patient with schizophrenia [21]. That patient was expected to have low compliance and thus remained sedated in an intensive care unit for five days after surgery with a Denhardt mouth gag [21]. Replantation of an amputated tongue portion is challenging, since the tongue is essentially a muscular organ. Another report has described necrosis after replantation of an amputated tongue portion that had remained ischemic for a long time [31]. Our patient (Case 1) brought the dry, necrotic amputated part of the tongue when she presented at the hospital. We did not replant it, because the degree of irreversible damage was likely to be too high. Despite the missing tissue, this patient appeared to be functioning well at one week after surgery. Her articulation and intelligibility were such that relatives could understand her speech over the telephone and she had no difficulty eating any type of food. The other patient (Case 2) did not keep a postoperative follow-up appointment one week after surgery. Therefore, her functional ability and wound condition remain unknown. Patients with psychiatric disorders are expected to have low compliance. Thus, successful outcomes should require intervention with the interdisciplinary cooperation of patients, relatives, healthcare providers, and a medical team that includes dentists as well as oral and maxillofacial surgeons.

Our patient had no continuous arterial bleeding from the tongue and developed no fatal systemic condition after the self-inflicted amputation. However, while people with NSSI do not intend to commit suicide, they might cause more harm than they intended, which could result in severe medical complications or death. Moreover, severe or prolonged self-injury might lead to desperation about lack of control over the causative issue, the self-destructive behavior, and its addictive nature. This might then escalate to true suicide attempts. Therefore, prompt intervention is important for NSSI in the oral cavity to avoid serious bleeding complications, infection, and the possibility of further self-mutilation. On the other hand, the characteristics of intraoral NSSI remain unclear, because it is rare. Tools for assessing NSSI epidemiology in community populations are few and are either limited in the scope of NSSI features assessed [32] or do not include intraoral NSSI. The NSSI Assessment Tool (AT) has been validated and is useful for determining prevalence, form, function, recency, and severity [33]. Future studies should investigate the features of intraoral NSSI using the NSSI-AT. Since the “wound locations” including the lips or tongue are evaluation items in the NSSI-AT, a detailed analysis might reveal relationships between the motivation to inflict intraoral NSSI.

## 4. Conclusion

We described two nonsuicidal patients who mutilated their tongues using edged tools after sleep disturbances. The prevalence of NSSI has recently increased, and concerns have been raised about the potential normalization of self-mutilation. Although such patients are rare, medical providers including dentists and oral surgeons should be aware of the increasing likelihood that a patient will present with a major NSSI seeking emergency treatment late at night.

## Figures and Tables

**Table 1:** Clinical reports of NSSI of the oral cavity inflicted using edged tools.

No.	Author	Year	Age	Sex	Tools	Lesion	Symptom	Psychological factors
1	Blanton et al.	1977	61	M	Knife	Teeth	Crown fracture	Unknown
2	McGrath et al.	1985	25	F	Knife	Gum	Ulcer	Unknown
3	Hong and Eun	2014	24	M	Scissors	Tongue	Amputation	Schizophrenia
4	Present case	2019	55	F	Knife	Tongue	Amputation	Depression
5	Present case	2019	69	F	Scissors	Tongue	Amputation	Schizophrenia
